# Efficacy of Neonatal HBV Vaccination on Liver Cancer and Other Liver Diseases over 30-Year Follow-up of the Qidong Hepatitis B Intervention Study: A Cluster Randomized Controlled Trial

**DOI:** 10.1371/journal.pmed.1001774

**Published:** 2014-12-30

**Authors:** Chunfeng Qu, Taoyang Chen, Chunsun Fan, Qimin Zhan, Yuting Wang, Jianhua Lu, Ling-ling Lu, Zhengping Ni, Fei Huang, Hongyu Yao, Jian Zhu, Jian Fan, Yuanrong Zhu, Zhiyuan Wu, Guoting Liu, Wenhong Gao, Mengya Zang, Dongmei Wang, Min Dai, Chu Chieh Hsia, Yawei Zhang, Zongtang Sun

**Affiliations:** 1 State Key Lab of Molecular Oncology, Cancer Institute/Hospital, Chinese Academy of Medical Sciences & Peking Union Medical College, Beijing, China; 2 Qidong Liver Cancer Institute, Qidong, Jiangsu Province, China; 3 National Office for Cancer Prevention and Control, Cancer Institute/Hospital, Chinese Academy of Medical Sciences, Beijing, China; 4 Department of Environmental Health Sciences, Yale School of Public Health, Yale University School of Medicine, New Haven Connecticut, United States of America; University of Texas Southwestern Medical Center, United States of America

## Abstract

**Background:**

Neonatal hepatitis B vaccination has been implemented worldwide to prevent hepatitis B virus (HBV) infections. Its long-term protective efficacy on primary liver cancer (PLC) and other liver diseases has not been fully examined.

**Methods and Findings:**

The Qidong Hepatitis B Intervention Study, a population-based, cluster randomized, controlled trial between 1985 and 1990 in Qidong, China, included 39,292 newborns who were randomly assigned to the vaccination group in which 38,366 participants completed the HBV vaccination series and 34,441 newborns who were randomly assigned to the control group in which the participants received neither a vaccine nor a placebo. However, 23,368 (67.8%) participants in the control group received catch-up vaccination at age 10–14 years. By December 2013, a total of 3,895 (10.2%) in the vaccination group and 3,898 (11.3%) in the control group were lost to follow-up. Information on PLC incidence and liver disease mortality were collected through linkage of all remaining cohort members to a well-established population-based tumor registry until December 31, 2013. Two cross-sectional surveys on HBV surface antigen (HBsAg) seroprevalence were conducted in 1996–2000 and 2008–2012. The participation rates of the two surveys were 57.5% (21,770) and 50.7% (17,204) in the vaccination group and 36.3% (12,184) and 58.6% (17,395) in the control group, respectively. Using intention-to-treat analysis, we found that the incidence rate of PLC and the mortality rates of severe end-stage liver diseases and infant fulminant hepatitis were significantly lower in the vaccination group than the control group with efficacies of 84% (95% CI 23%–97%), 70% (95% CI 15%–89%), and 69% (95% CI 34%–85%), respectively. The estimated efficacy of catch-up vaccination on HBsAg seroprevalence in early adulthood was 21% (95% CI 10%–30%), substantially weaker than that of the neonatal vaccination (72%, 95% CI 68%–75%). Receiving a booster at age 10–14 years decreased HBsAg seroprevalence if participants were born to HBsAg-positive mothers (hazard ratio [HR] = 0.68, 95% CI 0.47–0.97). Limitations to consider in interpreting the study results include the small number of individuals with PLC, participants lost to follow-up, and the large proportion of participants who did not provide serum samples at follow-up.

**Conclusions:**

Neonatal HBV vaccination was found to significantly decrease HBsAg seroprevalence in childhood through young adulthood and subsequently reduce the risk of PLC and other liver diseases in young adults in rural China. The findings underscore the importance of neonatal HBV vaccination. Our results also suggest that an adolescence booster should be considered in individuals born to HBsAg-positive mothers and who have completed the HBV neonatal vaccination series.

*Please see later in the article for the Editors' Summary*

## Abstract 2

In a 30-year follow-up of the Qidong Hepatitis B Intervention Study, Yawei Zhang and colleagues examine the effects of neonatal vaccination on liver diseases.

*Please see later in the article for the Editors' Summary*

## Editors' Summary

**Background:**

Hepatitis B is a life-threatening liver infection caused by the hepatitis B virus (HBV). HBV, which is transmitted through contact with the blood or other bodily fluids of an infected person, can cause both acute (short-term) and chronic (long-term) liver infections. Acute infections rarely cause any symptoms and more than 90% of adults who become infected with HBV (usually through sexual intercourse with an infected partner or through the use of contaminated needles) are virus-free within 6 months. However, in sub-Saharan Africa, East Asia, and other regions where HBV infection is common, HBV is usually transmitted from mother to child at birth or between individuals during early childhood and, unfortunately, most infants who are infected with HBV during the first year of life and many children who are infected before the age of 6 years develop a chronic HBV infection. Such infections can cause liver cancer, liver cirrhosis (scarring of the liver), and other fatal liver diseases. In addition, HBV infection around the time of birth can cause infant fulminant hepatitis, a rare but frequently fatal condition.

**Why Was This Study Done?:**

HBV infections kill about 780,000 people worldwide annually but can be prevented by neonatal vaccination—immunization against HBV at birth. A vaccine against HBV became available in 1982 and many countries now include HBV vaccination at birth followed by additional vaccine doses during early childhood in their national vaccination programs. But, although HBV vaccination has greatly reduced the rate of chronic HBV infection, the protective efficacy of neonatal HBV vaccination against liver diseases has not been fully examined. Here, the researchers investigate how well neonatal HBV vaccination protects against primary liver cancer and other liver diseases by undertaking a 30-year follow-up of the Qidong Hepatitis B intervention Study (QHBIS). This cluster randomized controlled trial of neonatal HBV vaccination was conducted between 1983 and 1990 in Qidong County, a rural area in China with a high incidence of HBV-related primary liver cancer and other liver diseases. A cluster randomized controlled trial compares outcomes in groups of people (towns in this study) chosen at random to receive an intervention or a control treatment (here, vaccination or no vaccination; this study design was ethically acceptable during the 1980s when HBV vaccination was unavailable in rural China but would be unethical nowadays).

**What Did the Researchers Do and Find?:**

The QHBIS assigned nearly 80,000 newborns to receive either a full course of HBV vaccinations (the vaccination group) or no vaccination (the control group); two-thirds of the control group participants received a catch-up vaccination at age 10–14 years. The researchers obtained data on how many trial participants developed primary liver cancer or died from a liver disease during the follow-up period from a population-based tumor registry. They also obtained information on HBsAg seroprevalence—the presence of HBsAg (an HBV surface protein) in the blood of the participants, an indicator of current HBV infection—from surveys undertaken in1996–2000 and 2008–2012. The researchers estimate that the protective efficacy of vaccination was 84% for primary liver cancer (vaccination reduced the incidence of liver cancer by 84%), 70% for death from liver diseases, and 69% for the incidence of infant fulminant hepatitis. Overall, the efficacy of catch-up vaccination on HBsAg seroprevalence in early adulthood was weak compared with neonatal vaccination (21% versus 72%). Notably, receiving a booster vaccination at age 10–14 years decreased HBsAg seroprevalence among participants who were born to HBsAg-positive mothers.

**What Do These Findings Mean?:**

The small number of cases of primary liver cancer and other liver diseases observed during the 30-year follow-up, the length of follow-up, and the availability of incomplete data on seroprevalence all limit the accuracy of these findings. Nevertheless, these findings indicate that neonatal HBV vaccination greatly reduced HBsAg seroprevalence (an indicator of current HBV infection) in childhood and young adulthood and subsequently reduced the risk of liver cancer and other liver diseases in young adults. These findings therefore support the importance of neonatal HBV vaccination. In addition, they suggest that booster vaccination during adolescence might consolidate the efficacy of neonatal vaccination among individuals who were born to HBsAg-positive mothers, a suggestion that needs to be confirmed in randomized controlled trials before booster vaccines are introduced into vaccination programs.

**Additional Information:**

Please access these websites via the online version of this summary at http://dx.doi.org/10.1371/journal.pmed.1001774.

- The World Health Organization provides a fact sheet about hepatitis B (available in several languages) and information about hepatitis B vaccination
- The World Hepatitis Alliance (an international not-for-profit, non-governmental organization) provides information about viral hepatitis, including some personal stories about hepatitis B from Bangladesh, Pakistan, the Philippines, and Malawi
- The UK National Health Service Choices website provides information about hepatitis B
- The not-for-profit British Liver Trust provides information about hepatitis B, including Hepatitis B: PATH B, an interactive educational resource designed to improve the lives of people living with chronic hepatitis B
- MedlinePlus provides links to other resources about hepatitis B (in English and Spanish)
- Information about the Qidong Hepatitis B intervention Study is available
- Chinese Center for Disease Control and Prevention provides links about hepatitis B prevention in Chinese

## Introduction

Infection with hepatitis B virus (HBV) in infancy or early childhood leads to a high rate of chronic HBV infection. Some perinatal infections from maternal HBV transmission may also cause fulminant hepatitis (FH) in infancy, acute hepatocyte necrosis leading to hepatic encephalopathy, and coagulopathy [1],[2]. Fulminant hepatitis B is a rare condition that develops in about 0.5% to 1% of cases [3]. The mortality rate of FH can be as high as 67% [2],[4]. The long-term major adverse outcomes of chronic HBV infection are primary liver cancer (PLC) and liver cirrhosis [5].

Worldwide, PLC is the fifth most commonly diagnosed cancer but the second leading cause of cancer death in men, the seventh most frequently diagnosed cancer, and the sixth most frequent cause of cancer deaths in women [6]. While aflatoxin, algal hepatotoxins in drinking water, betel nut chewing, diabetes mellitus, alcohol consumption, and tobacco use are also important risk factors for PLC development [7], controlling chronic HBV infection through universal neonatal HBV vaccination is instrumental in reducing incidences and mortalities of PLC and other liver diseases [8],[9]. In 1983, a WHO Scientific Group meeting was convened on the discussion of HBV vaccination for PLC prevention [10], and recommended to plan and initiate field trials of hepatitis B vaccination in populations where the prevalence of HBV infection and HCC are high. Many countries have since launched HBV vaccination programs based on the recommendations and accumulated scientific knowledge.

Studies from Taiwan, where data were analyzed from the Taiwan National Cancer Registry in birth cohorts born after the universal vaccination program as compared with the birth cohorts born before the program, have documented the effectiveness of HBV vaccination in reducing PLC incidence and mortality and FH [11]–[13]. A study among Alaska Native people of the United States reported the elimination of hepatocellular carcinoma (HCC) and acute hepatitis B in children 25 years after a hepatitis B immunization program [14]. Studies from Korea [15] and urban areas of China [16] have also reported a decreased incidence of PLC after the implementation of HBV vaccination programs. All these studies, based on the historical comparison of immunized and unimmunized cohorts at either the national or community level, support the hypothesis that HBV vaccination is associated with a reduced PLC risk. However, because of potential differences in baseline characteristics and in exposures to other risk factors between the immunized and historical comparison (unimmunized) birth cohorts, it is difficult to make the inference that the observed reduced risk of PLC was entirely attributable to HBV vaccination [11],[17],[18].

Qidong County, China, is a rural area with a high PLC incidence and mortality compared with China as a whole. The PLC incidence in Qidong was 79.6 per 100,000 in men and 23.1 per 100,000 in women during the period from 1978 to 2002 [19], and it was 28.15 per 100,000 in men and 9.31 per 100,000 in women in the China cancer registry, which covered 11 cities and counties from 1988 to 2005 [20]. The Qidong Hepatitis B Intervention Study (QHBIS), a population-based, cluster randomized, controlled trial of HBV vaccination conducted between 1983 and 1990 in Qidong, China, involving approximately 80,000 newborns who were randomly assigned to the vaccination or control groups [21],[22], provides us an unique opportunity to examine the efficacy of HBV vaccination against PLC and other liver diseases in young adulthood.

## Methods

### Overview of QHBIS

Study protocols (Text S1) were approved by the Ethical Committees of the Cancer Institute/Hospital, Chinese Academy of Medical Sciences (CI-CAMS) and the Qidong Liver Cancer Institute (QDLCI). The QHBIS [21],[22] was conducted in Qidong County, China, in 1983–1990, a window period when the vaccine was not available in any rural area of China. At the time, Qidong had a population of 1.1 million and approximately 13,000 births each year. The study has been described previously [21],[22] and was registered with Clinical Trials.gov number NCT00222664.

**Figure 1:**
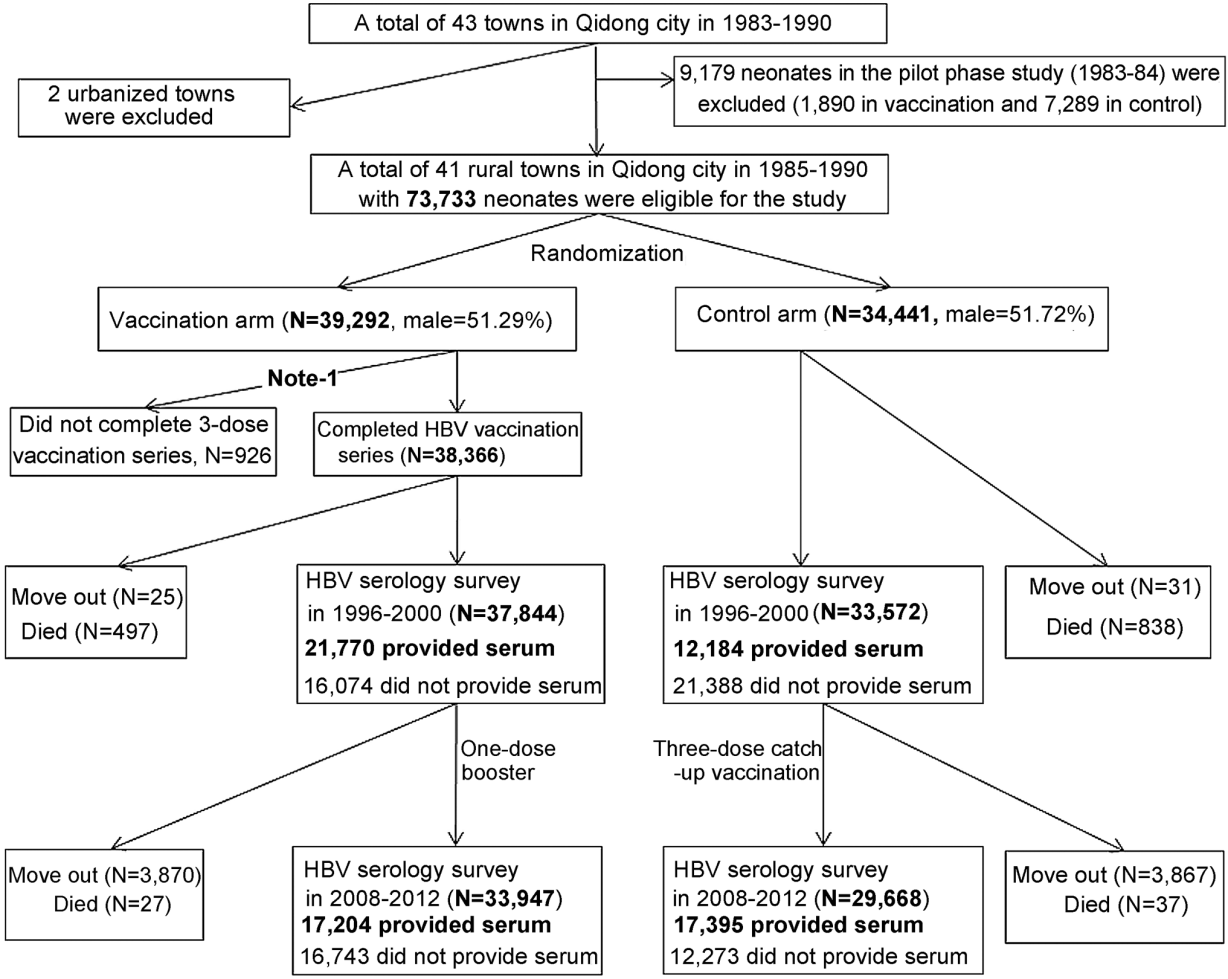
Flow-chart of the Qidong Hepatitis B Intervention Study. Note: 460 participants died within 6 months of age before completing the HBV vaccine series.

After a pilot phase in 1983–1984, which confirmed the feasibility, safety, and efficacy of HBV vaccination in a rural population, the main phase of the QHBIS was carried out in 1985–1990. The cluster randomized controlled trial included 41 rural towns (originally designated “communes”) across six clusters. The towns (as the basic unit) within each cluster allocated to HBV vaccination were randomly chosen by lottery under the supervision of the health representatives from each town (Figure 1). The PLC incidence rates in the vaccine covered towns were 59.2/10^5^ and 59.3/10^5^, respectively during 1985–1986 and 1987–1990, and the rates were 59.7/10^5^ and 59.9/10^5^, respectively during the same years in the control towns. Witnessed oral informed consent was obtained from eligible mothers in the vaccination coverage towns prior to birth delivery. All neonates in the vaccination towns (*n* = 39,292) were vaccinated regardless of maternal HBsAg status. The first dose (5 µg) of HBV vaccine was administered within 24 hours after birth, followed by two doses (5 µg/dose) at 1 and 6 months of age, respectively. A total of 38,366 (97.64%) participants completed the three-dose, 5 µg-plasma-derived HBV vaccination series, which were manufactured and donated by the Merck Company through the WHO. Maternal HBsAg status of each vaccinated participant was determined by reverse passive hemagglutination assay. Neonates in the control towns (*n* = 34,441) received neither vaccine nor placebo.

### Catch-up Vaccination

The HBV vaccines were exclusively controlled by the Chinese Centers for Disease Control and Prevention (CDC). During the study time period, plasma-derived or recombinant vaccine was not universally available. In 2000–2001, children born in Qidong County after 1986 in the control group were eligible for receiving a three-dose catch-up vaccination series, and those children in the vaccination group were eligible for receiving a one dose booster (adolescent booster). Each dose contained 10 µg recombinant vaccine (GlaxoSmithKline). No randomization was exercised for the catch-up and booster vaccinations (Text S2). A total of 23,368 (67.8%) individuals in the control group received the catch-up vaccination, and 28,988 (73.8%) children in the vaccination group received the booster.

### Liver Disease Outcomes

Each participant in the two groups was tracked by a study number linked to the mother's unique national identification number with detailed residence information recorded in the civil security administration system (HU-KOU in Chinese). When the participant reached age 16, this study number was linked to his/her own unique national identification number, and to his/her health insurance file. This system allowed us to notify participants of follow-up studies either themselves (16 years of age or older) or through a parent (under 16 years of age), as well as to collect valuable information pertaining to medical conditions and vital status.

A population-based cancer registry and department of vital statistics were established in QDLCI in 1972, collecting information on cancer incidence and mortality, as well as cause of death for all residents in Qidong County [23]. Data from this registry have been used by the International Agency for Research on Cancer to estimate cancer incidence in China [24]. The FH mortality (International Classification of Diseases [ICD]-9 code 570) and chronic liver disease mortality (ICD-9 code 571) were obtained from the vital statistics database, and PLC incidence (ICD-10 code C220 and C221) was obtained from the population-based cancer registry. Brain tumor incidence (ICD-10 code C710–C719) was also obtained from the population-based cancer registry as negative control. FH was defined as the development of hepatic encephalopathy and coagulopathy, increasing bilirubin levels with declining aminotransferase levels, within 8 weeks of disease onset in patients without known preexisting liver diseases. Children less than one month of age, and those with acute hepatic failure secondary to bacterial sepsis, drug intoxication, or total parenteral nutrition were not included [2],[4]. All deaths related to liver diseases after age three were diagnosed as acute-on-chronic liver failure (ACLF), which is defined as “acute hepatic insult manifesting as jaundice (serum bilirubin >5 mg/dl) and coagulopathy (international normalized ratio >1.5), complicated within 4 weeks by ascites and/or encephalopathy in a patient with previously diagnosed or undiagnosed chronic liver disease” [25]. Diagnostic criterion for PLC [26] did not change during the study period. The information pertaining to medical conditions and vital status among these participants were updated at age 16 years, when his/her own unique national identification card was issued, and yearly after that. All participants with FH, PLC, and ACLF identified in this population were verified through home visits and review of medical records.

### Serological Data

Upon receiving written consent, blood samples were collected from the study participants at age 10–11 years in 1996–2000 and at age 19–28 years in 2008–2012. A total of 21,770 (57.5%) individuals in the neonatal vaccination group and 12,184 (36.3%) individuals in the control group donated samples at age 10–11 years, and 17,204 (50.7%) in the vaccination group and 17,395 (58.6%) in the control group donated samples at age 19–28 years. All HBV serological markers were determined within 12 hours after blood sampling. Individuals with serum HBsAg-positivity were retested in six months. In 1996–2000, serum HBsAg was detected using ELISA reagents from Abbott Laboratories. In 2008–2012, serum HBsAg was detected using ELISA kits from Kehua Bioengineering Co., Ltd, Shanghai, China, with the detection limit for serum HBsAg of 0.5 ng/ml. For quality control, 10% of tested samples in 2008–2012 were re-tested using reagents from Roche Diagnostic GmbH in Cobas e-601 in CI-CAMS and the concordance rate of HBsAg serum positivity was 100%.

### Statistical Analysis

Person-years at risk were calculated from the date of birth to the first date of PLC diagnosis, death, moving out, or last follow-up (December 31, 2013). Cox proportional hazard models were employed to compute hazard ratio (HR) and 95% CIs, adjusting for cluster. For an HR expected to be less than 1, efficacy was calculated as (1 − HR) × 100%, and 95% CI was calculated using the lower and higher bound of HRs. Univariate analyses (Chi-squared test and ANOVA) were conducted to compare distributions of selected characteristics of the study population. Logistic regression models were employed to compare prevalence of HBsAg seropositivity between vaccination and control groups as well as to examine the associations between adulthood HBsAg positivity and infant gender, adolescence booster, and maternal HBsAg status. The efficacy of HBV vaccine against HBsAg seropositivity was estimated using odds ratios (ORs) and 95% CI. All analyses were performed using SAS 9.2.

## Results

### Incidence Rate of PLC and Mortality Rate of Severe End-Stage Liver Diseases

**Figure 2:**
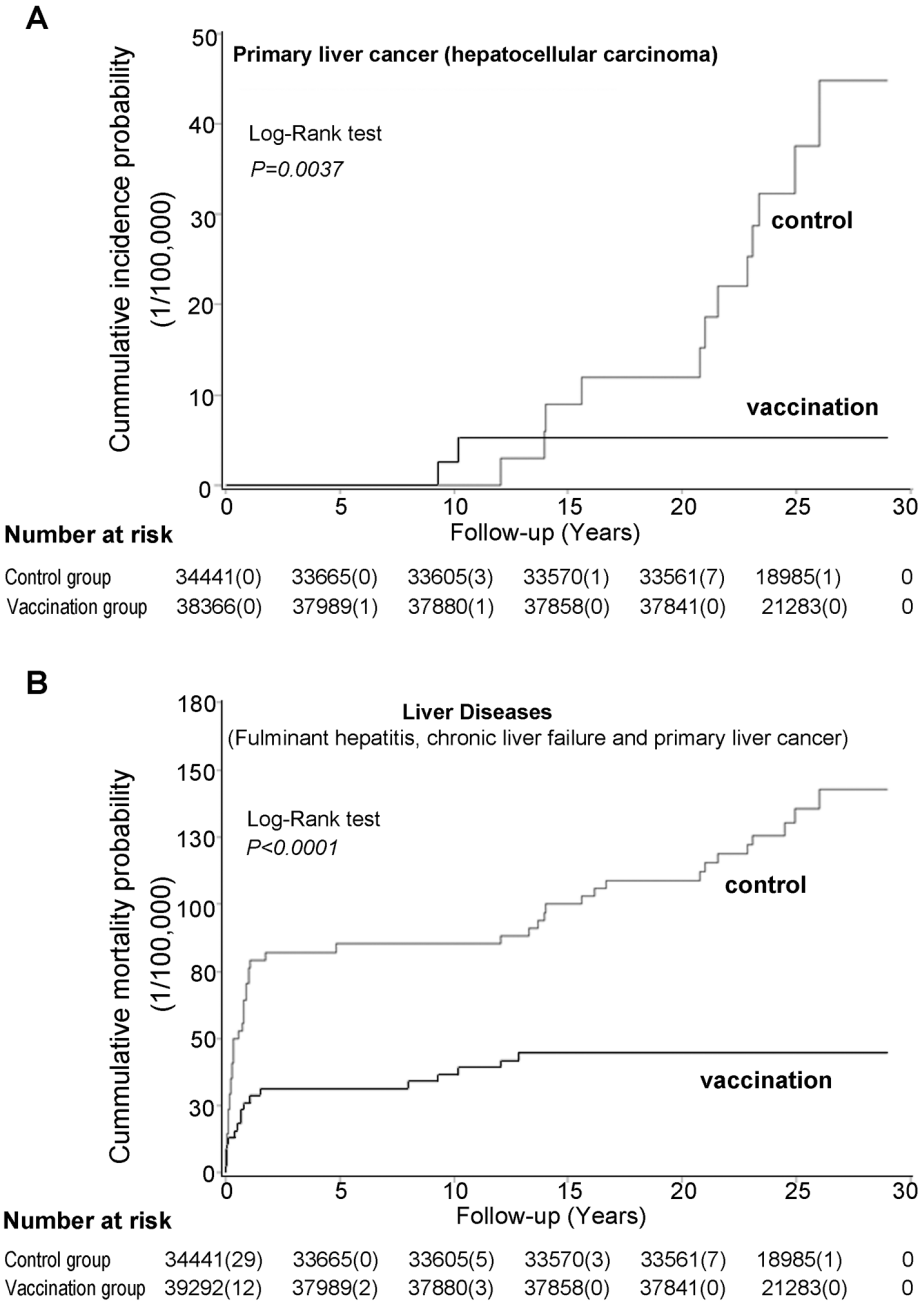
Cumulative incidence probability of primary liver cancer (A) and cumulative mortality probability of liver diseases (B) in the vaccination and control groups.

The median years of follow-up were 25.2 (interquartile [IQ] 23.7–26.7) and 25.0 (IQ 23.7–26.4) years in the control and vaccination groups, respectively. At the last follow-up, three participants (two males, one female) in the vaccination group and 14 participants (ten males, four females) in the control group were diagnosed with PLC (Figure 2; Table 1). Three of the individuals with PLC (one in the vaccination group and two in the control group) had hepatoblastoma. Among the remaining individuals with PLC, only two patients were histologically confirmed as HCC because all other patients took alternative therapies without available liver tissue for histological examination. One of the two patients in the vaccination group and all 12 individuals in the control group were HBsAg positive. Participants with PLC were only diagnosed in the control group when the individuals were aged 20 years or older. As a negative control, 11 participants (five in the vaccination group and six in the control group) were diagnosed with a brain tumor. The incidence rates between the vaccination group (0.52 per 100,000) and the control group (0.71 per 100,000) were similar (*p*>0.05).

**Table 1:** **Age distribution and diagnostic evidences of liver diseases in HBV vaccination group and control group.**

Diseases	Total Number of Diagnosed Individuals	Age (y)			Diagnostic Evidence							HBsAg		
		0–9	10–19	≥20	Medical Imaging				Histology	Biochemical	Death	+	−	N/A
					B	B + CT	CT	MRI	Detection	Detection	Certificate			
Primary liver cancer														
HCC														
Control group	12	1	3	8	0	5	6	1	2	12	0	12	0	0
Vaccination group	2	1	1	0	1	0	1	0	0	2	0	1	1	0
Hepatoblastoma														
Control group	2	2	0	0	2	0	0	0	0	0	0	0	0	2
Vaccination group	1	1	0	0	1	0	0	0	0	0	0	1	0	0
Acute-on-chronic liver failure														
Control group	6	1	4	1	0	0	0	0	0	6	0	4	0	2
Vaccination group	3	1	2	0	0	0	0	0	0	3	0	2	0	1
FH^a^														
Control group	28	28	N/A	N/A	0	0	0	0	0	0	28	0	0	28
Vaccination group	12	12	N/A	N/A	0	0	0	0	0	0	12	1	0	11
Brain tumor														
Control group	6	3	1	2	0	0	4	2	2	6	0	0	2	4
Vaccination group	5	1	2	2	0	0	4	1	1	5	0	0	4	1

a All deaths related to liver diseases after age three were classified as acute-on-chronic liver failure (ACLF).

B, B-ultrasonic scan; CT, computed tomography; MRI, magnetic resonance imaging; N/A, not available.

Hepatoblastoma was not associated with HBV [27], therefore these individuals were excluded from analysis for PLC and severe end-stage liver diseases as other reports did [11],[28]. The vaccination group had a significantly lower PLC incidence rate compared with the control group (HR = 0.16, 95% CI 0.03–0.77) with 84% (95% CI 23%-97%) protective efficacy against HBV-related PLC development before age 30 (Table 2).

**Table 2:** **Incidence rate and mortality rate of primary liver cancer and other liver diseases in vaccination and control groups.**

Liver Diseases	Person-Years under Observation	Number of Individuals Diagnosed	Incidence Rate	HR^a^ (95% CI)	*p*-Value	Protective Efficacy (95% CI)
Primary liver cancer (excluding hepatoblastoma)						
Control group	850,255	12	1.41	1	0.0224	84% (23%–97%)
Vaccination group	954,886	2	0.21	0.16 (0.03–0.77)		
Severe-end-stage liver diseases (primary liver cancer and acute-on-chronic liver failure)^b^						
Control group	850,258	17	2.00	1	0.0241	70% (15%-89%)
Vaccination group	954,886	5	0.52	0.30 (0.11–0.85)		
FH						
Control group	101,667	28	27.54	1	0.0024	69% (34%–85%)
Vaccination group	114,689	12	10.46	0.31 (0.15–0.66)		

a Adjusted for clusters.

b 13 of the 14 diagnosed PLC died by December 2013.

Among the 14 patients diagnosed with PLC, 11 in the control group and all in the vaccination group died within one year of diagnosis by December 2013. In addition, six participants (three males, three females) in the control group and three (one male, two females) in the vaccination group died of acute-on-chronic liver failure (ACLF). The mortality rate of severe end-stage chronic liver diseases, including PLC and chronic liver failure, was significantly lower in the vaccination group than in the control group (HR = 0.30, 95% CI 0.11–0.85), and the efficacy of neonatal vaccination against severe end-stage chronic liver diseases was 70% (95% CI 15%–89%) (Table 2).

Twelve participants (ten males, two females) in the vaccination group and 28 participants (13 males, 15 females) in the control group died of FH before age 3 years. The vaccination group had significantly lower FH mortality than the control group (HR = 0.31, 95% CI 0.15–0.66), and the efficacy of neonatal vaccination against FH mortality was 69% (95% CI 34%–85%) (Table 2).

### HBsAg Seroprevalence at Age 10–11 Years and Age 19–28 Years

Distributions of age and maternal age between the participants who donated blood samples and those who did not were similar among the vaccination group and control group at the two serological surveys, respectively (Table 3). Distribution of gender between those with and without blood samples was similar during the first survey; however, more males than females donated blood samples during the second survey because many female participants were in pregnancy or breast feeding. Maternal HBsAg status was only available for the vaccination group. Maternal HBsAg seroprevalence was similar between the participants who donated blood samples and those who did not in both surveys. The HBsAg seroprevalence was similar among the six centers during the two serological surveys, in both vaccination and control groups (Table 4).

**Table 3:** **Distributions of selected characteristics of the participants with and without serum samples during the two serologic surveys.**

Characteristics	Vaccination Group			Control Group		
	Serum	No Serum	*p-*Value	Serum	No Serum	*p-*Value
Serologic survey in 1996–2000						
Total number	21,770	16,074		12,184	21,388	
Age (years)						
9	475 (2.2%)	312 (1.9%)	0·24	274 (2.2%)	427 (2.0%)	0·13
10	10,855 (49.9%)	8,137 (50.6%)		6,182 (50.7%)	10,664 (49.9%)	
11	9,767 (44.9%)	7,140 (44.4%)		5,303 (43.5%)	9,535 (44.6%)	
12	673 (3.1%)	485 (3.0%)		425 (3.5%)	762 (3.6%)	
Gender						
Male	11,194 (51.4%)	8,217 (51.1%)	0·56	6,178 (50.7%)	10,936 (51.1%)	0·46
Female	10,576 (48.6%)	7,857 (48.9)%		6,007 (49.3%)	10,452 (48.9%)	
Maternal age (years)						
≤22	904 (4.2%)	733 (4.6%)	0·21	512 (4.2%)	869 (4.1%)	0·51
23	3,958 (18.2%)	2,893 (18.0%)		2,193 (18.0%)	3,873 (18.1%)	
24	4,871 (22.4%)	3,535 (22.0%)		2,758 (22.6%)	4,991 (23.3%)	
25	5,957 (27.4%)	4,449 (27.7%)		3,367 (27.6%)	5,780 (27.0%)	
26	4,071 (18.7%)	2,925 (18.2%)		2,258 (18.5%)	3,891 (18.2%)	
27–30	2,009 (9.2%)	1,539 (9.6%)		1,096 (9.0%)	1,984 (9.3%)	
Maternal HBsAg seroprevalence (%)	9·67%	9.71%	0.89	N/A	N/A	
Serologic survey in 2008–2012						
Total number	17,204	16,743		17,395	12,273	
Age (years)						
<20	2,480 (14.4%)	2,381 (14.2%)	0·48	2,309 (13.3%)	1,671 (13.6%)	0·28
20–23	4,845 (28.2%)	4,610 (27.5%)		4,597 (26.4%)	3,339 (27.2%)	
24–27	6,782 (39.4%)	6,707 (40.1%)		7,058 (40.6%)	4,899 (39.9%)	
≥28	3,097 (18.0%)	3,045 (18.2%)		3,431 (19.7%)	2,364 (19.3%)	
Gender						
Male	9,259 (53.8%)	8,310 (49.6%)	<0.0001	9,430 (54.2%)	6,105 (49.7%)	<0·0001
Female	7,945 (46.2%)	8,433 (50.4%)		7,965 (45.8%)	6,168 (50·3%)	
Maternal age (years)						
≤22	760 (4.4%)	657 (3.9%)	0.20	849 (4.9%)	622 (5.1%)	0·20
23	3,096 (18.0%)	3,083 (18.4%)		3,151 (18.1%)	2,189 (17.8%)	
24	3,735 (21.7%)	3,658 (21.8%)		3,693 (21.2%)	2,587 (21.1%)	
25	4,753 (27.6%)	4,553 (27.2%)		4,663 (26.8%)	3,371 (27.5%)	
26	3,412 (19.8%)	3,336 (19.9%)		3,453 (19.9%)	2,479 (20.2%)	
27–30	1,448 (8.4%)	1,456 (8.7%)		1,586 (9.1%)	1,025 (8.4%)	
Maternal HBsAg seroprevalence (%)	9.68%	9.66%	0.95	N/A	N/A	

N/A, not available.

**Table 4:** **HBsAg seroprevalence among different clusters in the surveys conducted in 1996–2000 and in 2008–2012.**

Clusters	Vaccination Group					Control Group				
	Blood (%)	Number Tested	Positive Number	Positive Rate (%)	*p-*Value	Blood (%)	Number Tested	Positive Number	Positive Rate (%)	*p-Value*
Serologic Survey in 1996–2000										
1	50.85	3,319	73	2.20	0.75	32.43	1,940	175	9.02	0.74
2	54.92	2,442	60	2.46		29.81	1,221	110	9.01	
3	57.48	3,939	91	2.31		40.56	2,543	224	8.81	
4	57.73	4,856	96	1.98		43.21	2,104	205	9.74	
5	64.01	6,360	134	2.11		46.02	1,786	167	9.35	
6	51.17	854	16	1.87		30.57	2,590	219	8.46	
Serologic Survey in 2008–2012										
1	50.74	2,972	49	1.65	0.75	49.24	2,782	172	6.18	0.63
2	48.21	1,930	39	2.02		57.17	2,083	146	7.01	
3	52.15	3,198	65	2.03		55.49	2,782	183	6.58	
4	50.29	3,588	61	1.70		64.83	2,684	189	7.04	
5	51.02	4,790	85	1.77		60.77	1,992	125	6.28	
6	50.73	726	16	2.20		63.85	5,072	356	7.02	

The HBsAg seropositive rates in the vaccination group were 2.16% at age 10–11, and 1.83% at 19–28 years, respectively, which were significantly lower than those in the control group (9.03% at age 10–11 and 6.73% at age 19–28, *p*<0.0001). The protection efficacy of neonatal vaccination against HBsAg seropositivity was 78% (95% CI 75%–80%) and 72% (95% CI 68%–75%), respectively (Table 5).

**Table 5:** **Neonatal and catch-up vaccination (administered at age 10–14 years) on HBsAg seroprevalence.**

Participant Group	Total Number	HBsAg Seropositivity		*p-*Value^a^	OR (95% CI)	Efficacy (95% CI)
	Tested	Number	Rate			
Age 10–11 years						
Control group	12,184	1,100	9.03%	<0.0001	1	78% (75%–80%)
Vaccination group	21,770	470	2.16%		0.22 (0.20–0.25)	
Age 19–28 years						
Control group	17,395	1,171	6.73%	<0.0001	1	72% (68%–75%)
Vaccination group	17,204	315	1.83%		0.28(0.25–0.32)	
Control group without catch-up vaccination	5,518	428	7.76%	0.0002	1	21% (10%–30%)
Control group with catch-up vaccination	11,877	743	6.26%		0.79 (0.70–0.90)	

Catch-up vaccination was not randomized.

a Chi-square tests.

Catch-up vaccination was administrated to 23,368 (67.8%) controls at age 10–14 years. HBsAg seropositive rate was 6.26% in those who received the catch-up vaccination, lower than those (7.76%) who did not receive the catch-up vaccination (*p* = 0.0002). The protection efficacy was 21% (95% CI 10%–30%), substantially weaker compared with neonatal vaccination (Table 5).

A total of 10,051 individuals in the vaccination group and 5,990 individuals in the control group participated in both serologic surveys (Table 6). No significant differences in HBsAg seroprevalence between the two surveys were found in the vaccination group (*p* = 0.5938) and the control group without catch-up vaccination (*p* = 0.1516). However, difference in HBsAg seroprevalence between the two surveys was observed in the control group with catch-up vaccination (*p* = 0.0008). The annual HBsAg seroclearance rates were 0.36%, 1.49%, and 1.09% in the vaccination group, control group with catch-up vaccination, and control group without catch-up vaccination, respectively.

**Table 6:** **HBsAg seropositivity and HBsAg annual seroclearance in the participants who participated in both serologic surveys.**

Participants	Number	HBsAg Seropositive		*p*-Value	Person-Years under Observation^a^	HBsAg Annual Seroclearance^b^
		Number	Rate (%)			
Vaccination group						
Age 10–11 years	10,051	184	1.83	0.5938	2,760	0.36%
Age 19–28 years		174	1.73			
Control group without catch-up vaccination						
Age 10–11 years	1,019	95	9.32	0.1516	1,655	1.09%
Age 19–28 years		77	7.56			
Control group with catch-up vaccination						
Age 10–11 years	4,971	420	8.45	0.0008	6,019	1.46%
Age 19–28 years		332	6.68			

a HBsAg-positive participants.

b HBsAg annual seroclearance rates were calculated by dividing the number of incident cases of HBsAg seroclearance by person-years of follow-up.

### Risk Factors Related to Vaccination Protection Failures in Adulthood

At adulthood, the HBsAg seropositive rate among those vaccinated as neonates was 1.85% in females and 1.81% in males; no differences in seropositivity of HBsAg (Table 7) or antibody against HBsAg (anti-HBs) (Table 8) were observed for gender. Vaccinated infants had an approximately 16-fold increased risk of being chronic HBsAg carriers in adulthood if they were born to HBsAg-positive mothers as compared with those born to HBsAg-negative mothers (OR = 15.94, 95% CI 12.63–20.12) (Table 7).

**Table 7:** **Risk factors of vaccination protection failures in adults who received neonatal vaccination.**

Factors	HBsAg Seropositivity			OR (95% CI)	
	Numbers	Percent	*p*-Value	Unadjusted	Adjusted^a^
Gender					
Female (*n* = 7,940)	147	1.85	0.9282	1.00	1.00
Male (*n* = 9,258)	168	1.81		0.98 (0.78–1.23)	1.01 (0.80–1.27)
Maternal HBsAg^b^					
Negative (*n* = 15,505)	124	0.80	<0.0001	1.00	1.00
Positive (*n* = 1,661)	191	11.50		16.12 (12.78–20.33)	15.94 (12.63–20.12)
Adolescence booster^c^					
No (*n* = 3,175)	66	2.08	0.2980	1.00	1.00
Yes (*n* = 14,024)	249	1.78		0.85 (0.65–1.12)	0.86 (0.65–1.14)
Maternal HBsAg (−)					
No-adolescence booster (*n* = 2,849)	19	0.67	0.3733	1.00	1.00
Yes-adolescence booster (*n* = 12,656)	105	0.83		1.25 (0.76–2.03)	1.25 (0.76–2.05)
Maternal HBsAg (+)					
No-adolescence booster (*n* = 318)	47	14.78	0.0260	1.00	1.00
Yes-adolescence booster (*n* = 1,343)	144	10.72		0.69 (0.49–0.99)	0.66 (0.46–0.95)

a Adjusted for the variables listed in the tables and clusters.

b Maternal HBsAg seropositive rate was 9.68% in vaccination group.

c Adolescence booster: 10 µg of recombinant HBV vaccine (GlaxoSmithKline) were given at age 10–14 years. It was not randomized.

**Table 8:** **Seroprevalence of anti-HBs among young adults in the neonatal vaccination group.**

Factors	Anti-HBs Seropositivity		
	Numbers	Percent	*p*-Value
Gender			
Female (*n* = 3,405)	1,454	42.70	0.9831
Male (*n* = 3,154)	1,346	42.68	
Maternal HBsAg^a^			
Negative (*n* = 5,856)	2,484	42.42	0.326
Positive (*n* = 685)	304	44.38	
Adolescence booster^b^			
No (*n* = 1,410)	459	32.55	<0.0001
Yes (*n* = 5,150)	2,341	45.46	
Maternal HBsAg (−)			
No-adolescence booster (*n* = 1,261)	411	32.59	<0.0001
Yes-adolescence booster (*n* = 4,595)	2,073	45.11	
Maternal HBsAg (+)			
No-adolescence booster (*n* = 146)	48	32.88	0.0016
Yes-adolescence booster (*n* = 539)	256	47.50	

a Maternal HBsAg seropositive rate was 10.47% in vaccination group.

b Adolescence booster: 10 µg of recombinant HBV vaccine (GlaxoSmithKline) were given at age 10–14 years. It was not randomized.

The anti-HBs positive rate in participants who received a booster at age 10–14 years was higher than those who did not receive a booster regardless of maternal HBsAg status (Table 8). Receiving the adolescence booster decreased HBsAg seroprevalence if participants were born to HBsAg-positive mothers (HBsAg positive rate 10.72% versus 14.78%, respectively, OR = 0.66, 95% CI 0.46–0.95) (Table 7).

## Discussion

Our study demonstrates that neonatal HBV vaccination has a significantly protective effect against PLC development in young adults. PLC was only diagnosed in participants in the control group but not in the vaccination group when the individuals reached 20 years or older. In addition, we have revealed a significant protective effect of neonatal HBV vaccination in decreasing the mortality rate of severe end-stage chronic liver diseases caused by chronic HBV infection. The protective efficacy of neonatal HBV vaccination was further supported by the measured reduction of HBsAg seroprevalence in young adults in the vaccination group as compared with those in the control group, although a majority of the participants in the control group received the catch-up vaccination in late childhood and early adolescence. Given that PLC and liver cirrhosis are mainly diagnosed in adults after age 40–45 years [5],[29], the study population is still young and incidence and deaths were very low. Although regression models suggested a significant difference, longer follow-up is warranted. More cases may be needed to fully verify this finding, as well as to understand the efficacy of HBV vaccination on the prevention of PLC and liver cirrhosis as this population ages.

Reports from several countries have documented decreases in HBsAg seroprevalence after launching HBV vaccination [8],[9]. It has been reported that the incidence rate of becoming a chronic HBsAg carrier is around 90% among individuals infected during the perinatal period, but it decreases as one ages [1]. Our study showed catch-up vaccination received at age 10–14 years reduced HBsAg seroprevalence in young adults with roughly 20% efficacy, compared with 72% efficacy of neonatal vaccination. This finding highlights the importance of HBV vaccination on neonates against chronic HBV infection in HBV highly endemic regions.

Vertical transmission is a major route for HBV infection in Asian countries and endemic areas; it accounts for about 40%–50% of HBsAg carriers in Taiwan and in mainland China. Horizontal transmission through close contact among children and family members is also a critical route for HBV infection [2],[30],[31]. Our study suggests that catch-up vaccination to children without neonatal vaccination who are younger than 15 years old and living in an HBV endemic area has a protective effect against HBV infection. Because the catch-up vaccination in our study was not randomized, future studies are needed to confirm the findings. Studies in China [32] and among the Alaska Native population [14],[33] suggest that catch-up vaccination to children and adolescents is cost-effective.

Spontaneous HBsAg seroclearance was frequently found in adults with an average annual clearance rate ranging from 1.15% to 2.26% in Chinese [34],[35] and 0.5% in Alaska Native populations in the United States [36]. Our study showed a relatively higher annual HBsAg seroclearance rate among individuals who received catch-up vaccination compared with those without a catch-up vaccination, suggesting that catch-up vaccination among children in endemic areas might play a role in the reduction of HBsAg seroprevalence. Again, the results need to be confirmed since the catch-up vaccination in our study was not randomized.

Neonatal hepatitis B vaccination-conferred antibodies against HBV surface antigens wane after 10 to 15 years [8]. In several recent studies, researchers reported that about one-quarter of neonatal vaccine recipients had lost their immune memory to the HBV vaccines when entering young adulthood [37],[38]. Because young adults may engage in risky behaviors that potentiate horizontal transmission of HBV as they become sexually active [31], the booster policy was recently recommended for consideration in highly endemic regions, such as some Asian countries [39]. Our current study showed that a booster at age 10–14 years might improve the neonatal vaccinee's immunological memory and potentially prevent HBV horizontal infection in young adults. Again, the results need to be confirmed since the booster vaccination in our study was not randomized.

Consistent with the literature, maternal HBsAg seropositive status is the most significant independent risk factor for HBV vaccination failure among neonates [8],[13],[40]. Recent studies have demonstrated that the risk of vaccination failure increased significantly in individuals born to mothers who were HBV e antigen (HBeAg) positive or who had high serum HBV-DNA levels [8],[41],[42]. Because few mothers in our study were tested for HBeAg status when they were enrolled in the 1980s and quantification for serum HBV-DNA has only been available in recent decades, we were unable to evaluate the effect of HBeAg status or HBV viral load on neonatal vaccination and the adolescence booster against HBV infection.

Currently, China's national HBV vaccination program recommends three doses of HBV vaccines (each dose contains either 10 µg vaccine from recombinant yeast or 20 µg vaccine from Chinese hamster oocytes) for all newborns. For neonates born to HBsAg-positive mothers, administration of hepatitis B immunoglobulin (HBIG) within 12 hours after birth combined with a full course of HBV vaccine has been recommended [43]. Since no participants in our study received HBIG, we were unable to evaluate the long-term efficacy of HBIG against HBV infection.

Although the HBsAg seroprevalence decreased dramatically in participants who received vaccine compared with individuals in the control group, the HBsAg seropositivity rate in the vaccinated population is still about 2%, which is quite high compared with other reports [8],[9]. Possible explanations include a low dose of the vaccines (5 µg vaccine of each dose) used, without administration of HBIG, and the low coverage of vaccination. When the trial started in the 1980s, less than 50% of the neonates were vaccinated [21]. With increased HBV vaccination coverage and administration of HBIG to neonates from HBsAg-positive mothers, the HBsAg seroprevalence dropped to 0.36% (three out of 823) in children aged 6–9 years in Qidong (Table S1).

Maternal HBV transmission to infants may cause acute or FH in infancy, especially from mothers who are HBsAg-positive but HBeAg-negative [2],[44]. A nationwide HBV immunization program successfully lowered the mortality of FH in Taiwan [12]. Our study also demonstrated that neonatal HBV vaccination can reduce FH mortality with a 69% efficacy. However, this result is limited by the fact that all cases of FH were clinically diagnosed without further laboratory examination and liver tissue histological confirmation.

Our study was built upon a large population-based, randomized, controlled trial, which produced comparable groups at baseline. This population-based cluster randomized controlled trial was initiated in the 1980s when the HBV vaccine was unavailable to the rural Chinese population. Although this study could not be conducted currently, it was considered ethically justifiable during the time period of randomization because the recombinant vaccine was not universally available in China. It is possible that certain liver cancer risk factors might become disproportionally distributed between the two groups during the 30-year follow-up. HBV and Aflatoxin were demonstrated as two major risk factors in hepatocarcinogenesis in Qidong before the 1990s [45],[46]. Urinary aflatoxin M1, a main soluble metabolite after aflatoxin intake, was undetectable in nearly all adults after 1990 because of a sharp transition in their primary dietary staple food source [17]. Therefore, contributions from aflatoxin to PLC in our study population was likely minimal. Other potential factors such as alcohol consumption, cigarette smoking, diabetes, and obesity have been linked to liver cancer [47]; however, there is no evidence that these factors play a major role in liver cancer among young adults. In addition, two groups had similar incidence rates of a brain tumor occurring during the follow-up period, which further demonstrated that the two groups were comparable. Since a large proportion of our study participants in the control group received catch-up vaccination, it is likely that the observed protective efficacy of HBV neonatal vaccination against PLC and other chronic liver diseases is underestimated.

Qidong has had a well-established, population-based cancer registry since 1972, and its population has remained relatively stable [23]. Although in the past two decades about 100,000 residents have worked outside of Qidong each year, their census registration and information related to health care is still kept in Qidong [23]. Approximately 10% of the participants in both the vaccination and control groups moved out after age 18; the number of missing cases due to loss to follow-up is likely minimal.

Several other limitations should also be considered when interpreting the study results. PLC was histologically confirmed only in two patients. An early study conducted in the same area reported that more than 98% of the individuals diagnosed with PLC had HCC [17]. HBV infectious markers were found in 99% of the HCC patients in Qidong [46]. Given that all individuals in our study with PLC, except one, were HBsAg-positive, it is less likely that the participants diagnosed with PLC were HBV unrelated. Although the information on HBV anti-viral treatment is unavailable, the number of participants who received anti-viral treatment is likely minimal. Because the HBV anti-viral treatment was initiated in 2001 in Qidong, and only to adult patients with HBeAg-positive or overt chronic hepatitis B, none of the participants in our study were eligible for receiving anti-viral treatments. In 2007 HBV anti-viral therapy was made available to all chronic hepatitis B patients with serum HBV more than 10^4^ IU/ml in Qidong. However, very few patients received this therapy because it was not covered by health insurance and was likely unaffordable to the farmers in Qidong. Therefore, lack of information on anti-viral treatment was unlikely play a major role in our study results. Another potential limitation was that only about half of the individuals agreed to participate in serological surveys. Although individuals who participated or did not participate in the two surveys were similar, future studies are warranted to confirm the results generated from the surveys. The study generalizability to HBV prevention in populations with very low prevalence of HBsAg might be limited. In the United States, HBV remains an important cause of acute and chronic liver disease; a comprehensive vaccination strategy that includes catch-up vaccination of adolescents as well as adults in high-risk groups is recommended to protect people who remain at risk of HBV infection [48],[49]. Because HBV is highly endemic and most infection occurs prenatally in low- and middle-income countries, universal vaccination protecting against HBV in newborns is necessary and cost-effective [8],[50].

In conclusion, our study has provided novel evidence that neonatal HBV vaccination can significantly reduce the risk of PLC and other chronic liver diseases in young adulthood. Our study also suggests that an adolescent booster should be considered for individuals who were born to HBsAg-positive mothers and were HBsAg-negative in order to consolidate vaccination efficacy.

## Supporting Information

Table S1 **HBsAg seroprevalence in Qidong children aged 6–9 years, 2009.**
(DOCX)

Checklist S1 **CONSORT checklist.**
(DOCX)

Text S1 **The Qigong Hepatitis B Intervention Study protocol.**
(DOCX)

Text S2 **Catch up vaccination study protocol.**
(DOCX)

## Abbreviations

FH: fulminant hepatitis
HBV: hepatitis B virus
HBsAg: hepatitis B virus surface antigen
HCC: hepatocellular carcinoma
HR: hazard ratio
OR: odds ratio
PLC: primary liver cancer
QHBIS: Qidong Hepatitis B Intervention Study

## References

[1] Hyams KC (1995) Risks of chronicity following acute hepatitis B virus infection: a review. Clin Infect Dis 20: 992–1000. 7795104 10.1093/clinids/20.4.992

[2] Chang MH (2007) Hepatitis B virus infection. Semin Fetal Neonatal Med 12: 160–167. 17336170 10.1016/j.siny.2007.01.013

[3] Finelli L, Bell BP (2011) Chapter 4: Hepatitis B. VPD survellance manual, 5th edition: 4-1-13.

[4] Chang MH, Lee CY, Chen DS, Hsu HC, Lai MY (1987) Fulminant hepatitis in children in Taiwan: the important role of hepatitis B virus. J Pediatr 111: 34–39. 3110389 10.1016/s0022-3476(87)80338-4

[5] McMahon BJ (2009) The natural history of chronic hepatitis B virus infection. Hepatology 49: S45–55. 19399792 10.1002/hep.22898

[6] Jemal A, Bray F, Center MM, Ferlay J, Ward E, et al (2011) Global cancer statistics. CA Cancer J Clin 61: 69–90. 21296855 10.3322/caac.20107

[7] Poon D, Anderson BO, Chen LT, Tanaka K, Lau WY, et al (2009) Management of hepatocellular carcinoma in Asia: consensus statement from the Asian Oncology Summit 2009. Lancet Oncol 10: 1111–1118. 19880065 10.1016/S1470-2045(09)70241-4

[8] Chen DS (2009) Hepatitis B vaccination: the key towards elimination and eradication of hepatitis B. J Hepatol 50: 805–816. 19231008 10.1016/j.jhep.2009.01.002

[9] Zanetti AR, Van Damme P, Shouval D (2008) The global impact of vaccination against hepatitis B: a historical overview. Vaccine 26: 6266–6273. 18848855 10.1016/j.vaccine.2008.09.056

[10] Zuckerman AJ, Sun TT, Linsell A, Stjernsward J (1983) Prevention of primary liver cancer-report on a meeting of a W.H.O. Scientific Group. Lancet 1: 463–465. 6131180

[11] Chang MH, You SL, Chen CJ, Liu CJ, Lee CM, et al (2009) Decreased incidence of hepatocellular carcinoma in hepatitis B vaccinees: a 20-year follow-up study. J Natl Cancer Inst 101: 1348–1355. 19759364 10.1093/jnci/djp288

[12] Chiang CJ, Yang YW, You SL, Lai MS, Chen CJ (2013) Thirty-year outcomes of the national hepatitis B immunization program in Taiwan. JAMA 310: 974–976. 24002285 10.1001/jama.2013.276701

[13] Chien YC, Jan CF, Chiang CJ, Kuo HS, You SL, et al (2014) Incomplete hepatitis B immunization, maternal carrier status, and increased risk of liver diseases: a 20-year cohort study of 3.8 million vaccinees. Hepatology 60: 125–132. 24497203 10.1002/hep.27048

[14] McMahon BJ, Bulkow LR, Singleton RJ, Williams J, Snowball M, et al (2011) Elimination of hepatocellular carcinoma and acute hepatitis B in children 25 years after a hepatitis B newborn and catch-up immunization program. Hepatology 54: 801–807. 21618565 10.1002/hep.24442

[15] Gwack J, Park SK, Lee EH, Park B, Choi Y, et al (2011) Hepatitis B vaccination and liver cancer mortality reduction in Korean children and adolescents. Asian Pac J Cancer Prev 12: 2205–2208. 22296357

[16] Wu QJ, Vogtmann E, Zhang W, Xie L, Yang WS, et al (2012) Cancer incidence among adolescents and young adults in urban Shanghai, 1973-2005. PLoS One 7: e42607. 22880052 10.1371/journal.pone.0042607 PMC3411830

[17] Sun Z, Chen T, Thorgeirsson SS, Zhan Q, Chen J, et al (2013) Dramatic reduction of liver cancer incidence in young adults: 28 year follow-up of etiological interventions in an endemic area of China. Carcinogenesis 34: 1800–1805. 23322152 10.1093/carcin/bgt007 PMC3731800

[18] Chen JG, Kensler TW (2013) Changing rates for liver and lung cancer in Qidong, China. Chem Res Toxicol 27: 3–6. 24215631 10.1021/tx400313j PMC3946948

[19] Chen JG, Zhu J, Parkin DM, Zhang YH, Lu JH, et al (2006) Trends in the incidence of cancer in Qidong, China, 1978-2002. Int J Cancer 119: 1447–1454. 16596645 10.1002/ijc.21952

[20] Gao J, Xie L, Chen WQ, Zhang SW, Wu QJ, et al (2013) Rural-urban, sex variations, and time trend of primary liver cancer incidence in China, 1988-2005. Eur J Cancer Prev 22: 448–454. 23411745 10.1097/CEJ.0b013e32835de82a

[21] Sun Z, Ming L, Zhu X, Lu J (2002) Prevention and control of hepatitis B in China. J Med Virol 67: 447–450. 12116043 10.1002/jmv.10094

[22] Sun Z, Zhu Y, Stjernsward J, Hilleman M, Collins R, et al (1991) Design and compliance of HBV vaccination trial on newborns to prevent hepatocellular carcinoma and 5-year results of its pilot study. Cancer Detect Prev 15: 313–318. 1665400

[23] Chen JG (2013) Qidong Cancer Registration System Cancer Cancer in Qidong, China. Chen JG, editor. Beijing: Military Medical Science Press. pp9-23.

[24] Parkin DM, Whelan SL, Ferlay J, Teppo L, Thomas DB (2002) Cancer incidence in five continents, vol. VIII. Sci Publ number 155. Lyon: IARC. pp 212–231.

[25] Sarin SK, Kumar A, Almeida JA, Chawla YK, Fan ST, et al (2009) Acute-on-chronic liver failure: consensus recommendations of the Asian Pacific Association for the study of the liver (APASL). Hepatol Int 3: 269–282. 19669378 10.1007/s12072-008-9106-x PMC2712314

[26] Wu M, Shen F (2010) Liver cancer. 3rd edition.. Beijing: Peking University Medical Press pp.327.

[27] Wiwanitkit V (2005) Hepatitis virus B is not a risk factor in hepatoblastoma patients. Asian Pac J Cancer Prev 6: 213–214. 16101336

[28] Chang MH, Shau WY, Chen CJ, Wu TC, Kong MS, et al (2000) Hepatitis B vaccination and hepatocellular carcinoma rates in boys and girls. JAMA 284: 3040–3042. 11122592 10.1001/jama.284.23.3040

[29] Kensler TW, Qian GS, Chen JG, Groopman JD (2003) Translational strategies for cancer prevention in liver. Nat Rev Cancer 3: 321–329. 12724730 10.1038/nrc1076

[30] Xia GL, Liu CB, Cao HL, Bi SL, Zhan MY, et al (1996) Prevalence of hepatitis B and C virus infections in the general Chinese population: results from a nationwide cross-sectional seroepidemiologic study of hepatitis A, B,C, D and E virus infections in China, 1992. Int Hepatol Commun 5: 12.

[31] Zhang HW, Yin JH, Li YT, Li CZ, Ren H, et al (2008) Risk factors for acute hepatitis B and its progression to chronic hepatitis in Shanghai, China. Gut 57: 1713–1720. 18755887 10.1136/gut.2008.157149 PMC2582333

[32] Hutton DW, So SK, Brandeau ML (2010) Cost-effectiveness of nationwide hepatitis B catch-up vaccination among children and adolescents in China. Hepatology 51: 405–414. 19839061 10.1002/hep.23310 PMC3245734

[33] Harpaz R, McMahon BJ, Margolis HS, Shapiro CN, Havron D, et al (2000) Elimination of new chronic hepatitis B virus infections: results of the Alaska immunization program. J Infect Dis 181: 413–418. 10669320 10.1086/315259

[34] Chu CM, Liaw YF (2007) HBsAg seroclearance in asymptomatic carriers of high endemic areas: appreciably high rates during a long-term follow-up. Hepatology 45: 1187–1192. 17465003 10.1002/hep.21612

[35] Liu J, Yang HI, Lee MH, Lu SN, Jen CL, et al (2010) Incidence and determinants of spontaneous hepatitis B surface antigen seroclearance: a community-based follow-up study. Gastroenterology 139: 474–482. 20434450 10.1053/j.gastro.2010.04.048

[36] Yalcin K, Acar M, Degertekin H (2003) Specific hepatitis B vaccine therapy in inactive HBsAg carriers: a randomized controlled trial. Infection 31: 221–225. 14562945 10.1007/s15010-003-3187-1

[37] Lu CY, Chiang BL, Chi WK, Chang MH, Ni YH, et al (2004) Waning immunity to plasma-derived hepatitis B vaccine and the need for boosters 15 years after neonatal vaccination. Hepatology 40: 1415–1420. 15565627 10.1002/hep.20490

[38] Zhu CL, Liu P, Chen T, Ni Z, Lu LL, et al (2011) Presence of immune memory and immunity to hepatitis B virus in adults after neonatal hepatitis B vaccination. Vaccine 29: 7835–7841. 21816197 10.1016/j.vaccine.2011.07.098

[39] Romano L, Carsetti R, Tozzi AE, Mele A, Zanetti AR (2014) Chronic hepatitis B infection in adolescents vaccinated at birth: an alarm bell in favor of the need for a booster? Hepatology 59: 349. 23695813 10.1002/hep.26458

[40] Ni YH, Huang LM, Chang MH, Yen CJ, Lu CY, et al (2007) Two decades of universal hepatitis B vaccination in taiwan: impact and implication for future strategies. Gastroenterology 132: 1287–1293. 17433322 10.1053/j.gastro.2007.02.055

[41] Wen WH, Chang MH, Zhao LL, Ni YH, Hsu HY, et al (2013) Mother-to-infant transmission of hepatitis B virus infection: significance of maternal viral load and strategies for intervention. J Hepatol 59: 24–30. 23485519 10.1016/j.jhep.2013.02.015

[42] Lu L, Chen B, Wang J, Wang D, Ji Y, et al (2014) Maternal transmission risk and antibody levels against hepatitis B virus e antigen in pregnant women. Int J Infect Dis 28: 41–4. 25245000 10.1016/j.ijid.2014.07.028

[43] Lu FM, Zhuang H (2009) Management of hepatitis B in China. Chin Med J (Engl) 122: 3–4. 19187608

[44] Beath SV, Boxall EH, Watson RM, Tarlow MJ, Kelly DA (1992) Fulminant hepatitis B in infants born to anti-HBe hepatitis B carrier mothers. BMJ 304: 1169–1170. 1392798 10.1136/bmj.304.6835.1169 PMC1882113

[45] Sun Z, Lu P, Gail MH, Pee D, Zhang Q, et al (1999) Increased risk of hepatocellular carcinoma in male hepatitis B surface antigen carriers with chronic hepatitis who have detectable urinary aflatoxin metabolite M1. Hepatology 30: 379–383. 10421643 10.1002/hep.510300204

[46] Ming L, Thorgeirsson SS, Gail MH, Lu P, Harris CC, et al (2002) Dominant role of hepatitis B virus and cofactor role of aflatoxin in hepatocarcinogenesis in Qidong, China. Hepatology 36: 1214–1220. 12395332 10.1053/jhep.2002.36366

[47] Chuang SC, La Vecchia C, Boffetta P (2009) Liver cancer: descriptive epidemiology and risk factors other than HBV and HCV infection. Cancer Lett 286: 9–14. 19091458 10.1016/j.canlet.2008.10.040

[48] Mast EE, Mahoney FJ, Alter MJ, Margolis HS (1998) Progress toward elimination of hepatitis B virus transmission in the United States. Vaccine 16 Suppl: S48-51 10.1016/s0264-410x(98)00294-1 9915035

[49] Ramachandran S, Purdy MA, Xia GL, Campo DS, Dimitrova ZE, et al. (2014) Recent population expansions of hepatitis B virus in the United States. J Virol. In press. 10.1128/JVI.01594-14 PMC4249146 25187549

[50] Arevalo JA, Washington AE (1988) Cost-effectiveness of prenatal screening and immunization for hepatitis B virus. JAMA 259: 365–369. 2961895

