# Supplementary material for: Efficacy of Neonatal HBV Vaccination on Liver Cancer and Other Liver Diseases over 30-Year Follow-up of the Qidong Hepatitis B Intervention Study: A Cluster Randomized Controlled Trial
Source: PLoS Med. 2014 Dec 30;11(12):e1001774. doi: 10.1371/journal.pmed.1001774 (PMC4280122; doi:10.1371/journal.pmed.1001774)
Supplement: Table S1 — HBsAg seroprevalence in Qidong children aged 6–9 years, 2009. (DOCX) [file pmed.1001774.s001.docx]

TableS1. HBsAg seroprevalence in Qidong children aged 6-9 years, 2009

|  |  | No. tested | HBsAg Positive No. | HBsAg Positive rate |
| --- | --- | --- | --- | --- |
| Total number | | 823 | 3 | 0•36% |
| Year born | |  |  |  |
|  | 2000 | 92 | 0 | 0•00% |
|  | 2001 | 306 | 2 | 0•65% |
|  | 2002 | 314 | 0 | 0•00% |
|  | 2003 | 111 | 1 | 0•90% |
| Gender | |  |  |  |
|  | Male | 411 | 3 | 0•73% |
|  | Female | 412 | 0 | 0•00% |
