# Supplementary material for: Efficacy of Neonatal HBV Vaccination on Liver Cancer and Other Liver Diseases over 30-Year Follow-up of the Qidong Hepatitis B Intervention Study: A Cluster Randomized Controlled Trial
Source: PLoS Med. 2014 Dec 30;11(12):e1001774. doi: 10.1371/journal.pmed.1001774 (PMC4280122; doi:10.1371/journal.pmed.1001774)
Supplement: Text S1 — The Qigong Hepatitis B Intervention Study protocol. (DOCX) [file pmed.1001774.s003.docx]

Text S1: The Qigong Hepatitis B Intervention Study

Study Protocol

Assessment of eligibility:

Qidong County of Jiangsu Province of China is located on the north shore of the Yangtze River opposite to Shanghai. Its population is about 1·1 million and the majority of the local inhabitants are farmers. The annual birth is approximately 13,000. There are 43 communes (GONG-SHE in Chinese, it is equal to town, XIANG in Chinese). The two communes, in which the inhabitants have somehow similar living environment as the urban residents, will be excluded from the study. In each town, there is a local hospital called commune hospital. Nearly all the newborns were the first born a consequence of family planning. The mother's ages were between 22- 30 years.

A. Pilot Study

The pilot study was scheduled to conduct in 1983-1984. A 2×2 randomized factorial pilot study was conducted inrandomly selected (selected by lottery under the supervision of the health representatives from each town)8 communes between September 1983 and July 1984 to compare the protection provided by different doses of vaccine (5 μg or 2.5 μg given an 0, 1 and 6 months of age) and by hepatitis B immune globulin (HBIG: 110 IU given at birth) in infants of carrier (HBsAg / HBeAg+ve) mothers. It was concluded (Sun TT et al al: J. Cell. Phys. 1986; Supp 4: 83-90)[[1](#_ENREF_1)] that: (a) that there was little difference in the degree of protection provided during the first year by 2.5 μg or by 5 μg doses, but that longer follow-up would be needed to see whether this protection was maintained, and whether a booster dose would be required; and (b) that the addition of HBIG did not improve the degree of protection, and might even reduce it when added to low-dose vaccine regimens. In analysis of the results from pilot study, a total of 406 infants fromone control commune was a volunteer, not randomly allocated. On the basis of this pilot study, and experience from other studies, a main study of HBV vaccination started in 41Qidongrural towns in 1985-1990.

B. Conduct the Main Study in Qidong

The main phase study is scheduled to conduct in 1985-1990. The two communes, in which the inhabitants have somehow similar living environment as the urban residents, should be excluded from the main phase study. All the 41rural communes will berecruited for the study. The communes (as the basic unit) allocated to HBV vaccination are randomly chosen by lottery under the supervision of the health representatives from each commune. The randomization will be done twice, one is in January of 1985 and one in January of 1987. The recruitment will be stopped when the vaccine is available in any part of the rural areas. The first randomization will cover half of the recruited towns(20 towns), and the second randomization will increase 5-6 more towns (25-26 towns),which will be randomly selected among the left control towns, with increased vaccine supplements.

All neonates in the vaccination communes are vaccinated regardless of maternal HBsAg status without charge for the vaccines. Infants in the vaccination communes will be vaccinated at birth (or, at most, up to 3 days of age), at 1 month and at 6 months, while infants in control communes will not be vaccinated, also nor placebo. Almost all women go to the commune hospitals for their delivery, so that in most cases it is a relatively simple matter to obtain a maternal blood sample just prior to delivery (for HBsAg and HBeAg status) and to vaccinate the infant soon after delivery in the vaccine-allocated communes. When delivery occurs outside the mother’s commune hospital (for example, at home or in another commune) the hospital obstetrician or barefoot doctor attempts to visit the mother as soon as possible after birth to give the first dose of vaccination. All mothers in the vaccine communes will then be given appointment cards with the dates they are to return for the 2nd and 3rd vaccinations of their infants. If a mother does not return for either vaccination then the obstetrician would visit the family home on the next day in order to give the injection. Vaccination of infants born in “vaccine” commune of mothers who live in “no vaccine” communes will be avoided (and even if the first vaccination is given, the 2nd and 3rd are not).

Vaccine ampoules (0.5ml at 10μg/ml concentration) will be supplied to “vaccine” communes by the Qidong Liver Cancer Institute (QDLCI) only after receipt of a written request from the director of the commune hospital. This will provide a check that all vaccine supplied will be used in the study. The vaccine will be kept in a 4 °C refrigerator provided for the study and placed near to the delivery room.

In both “vaccine” and “no vaccine” communes a master record will be kept for each birth, along with details of any blood analyses and vaccinations given in the hospitals (similar records will also be kept in the QDLCI). In addition, a separate card will be completed for each mother, with similar information kept on it, for use in the outpatient clinics. Every ten days or so details of all births and all vaccinations will be sent by each commune hospital to the QDLCI using the QDLCI messengers. These details will then be transferred by the study clerk to the QDLCI master records, and the 10-daily forms collected into monthly bundles, to be stored with a summary activity analysis.

All maternal and infant blood samples will be stored in the study refrigerator in the commune hospitals along with their respective request forms until they are collected (daily except Sunday) by the 6 full-time QDLCI messengers. The maternal blood samples will be routinely assayed in the QDLCI Virology department for HBsAg using RPHA (Dr. Lu Jian-hua) and for HBsAg, HBeAg and anti-HBs using Abbott radioimmuno-assay kits in Beijing Cancer Institute (Dr. Sun Tsung-tang). The results of blood analyses will be recorded on the request forms and sent back to the commune hospitals for their records. The QDLCI will keep a record of these results in the Virology department for each commune and these data will then be transferred by the study clerk to the master records in the QDLCI. Any remaining blood will then be stored in plastic tubes in a -30 °C freezer in the Virology department with back-up power supply and temperature alarms in order to avoid high summer temperatures and/or power cuts. This will be particularly important for the blood samples taken from infants at about 5 years of age to be used for the assessment of the efficacy of vaccination. Samples will not be allowed to thaw during storage; thawing particularly affects the antibody levels, which will be reduced by about one-third each time the samples are thawed and re-frozen

The study will be carefully monitored by the investigators in the QDLCI and by the commune hospital doctors, with reliable and detailed records being kept in the institute and in the hospitals. At approximately monthly intervals each commune hospital will be visited by one of the QDLCI researchers to sort out any difficulties and to compare these two sets of records for any major discrepancies.

In order to check the comparability of the vaccine-allocated communes and the control-allocated communes, the numbers of births should be enumerated in all 41 communes during the study period. The registry of cancers and deaths can then be used to follow all individuals born in this period. Long-term follow-up in these children will be obtained through the QDLCI registry of all cancers and deaths occurring throughout Qidong County. These will be filed separately for each commune and provide the diagnosis (cancer site or cause of death) and the evidence supporting the diagnosis. For all reported liver cancers, further investigations will be carried out to confirm or refute the diagnosis.

Reference:

1. Sun TT, Chu YR, Ni ZQ, Lu JH, Huang F, et al. (1986) A pilot study on universal immunization of newborn infants in an area of hepatitis B virus and primary hepatocellular carcinoma prevalence with a low dose of hepatitis B vaccine. J Cell Physiol Suppl 4: 83-90.
