# Supplementary material for: Efficacy of Neonatal HBV Vaccination on Liver Cancer and Other Liver Diseases over 30-Year Follow-up of the Qidong Hepatitis B Intervention Study: A Cluster Randomized Controlled Trial
Source: PLoS Med. 2014 Dec 30;11(12):e1001774. doi: 10.1371/journal.pmed.1001774 (PMC4280122; doi:10.1371/journal.pmed.1001774)
Supplement: Text S2 — Catch up vaccination study protocol. (DOCX) [file pmed.1001774.s004.docx]

Text S2: Catch up vaccination

In June 2000, the Qidong Center for Disease Control and Prevention (CDC) issued a Notification (File No. 2000-010) regarding on HBV catch-up vaccination and booster. Translations from original file are listed below:

Date: June 28, 2000

Subject: Immunization against HBV Infection

From: Qidong CDC

To: All town’s hospital in Qidong

Protocol:

1). Target population: All children who were born in 1986-1991 will receive HBV vaccination.

2). Vaccination: 0.5 ml (at 20μgg/ml concentration) vaccines, intramuscular injection.

For children who were not vaccinated: administer 3 doses at 0,1,6 month. The 1^st^ and 2^nd^ doses are given in July and August, 2000.

For children who were vaccinated previously: administer 1 dose between July and August 2000.

3). Fill in the vaccination card for each of the children.
